# Experiences and perceptions on antiretroviral therapy adherence and non-adherence: a scoping review of young people living with HIV in sub-Saharan Africa

**DOI:** 10.1186/s12889-025-22579-6

**Published:** 2025-04-17

**Authors:** Tamlyn Carmin Seunanden, Nothando Ngwenya, Janet Seeley

**Affiliations:** 1 School of Nursing and Public Health, Discipline of Public Health Medicine, University of KwaZulu-Natal, Durban, South Africa; 2 Africa Health Research Institute, KwaZulu-Natal, Durban, South Africa; 3 Department of Global Health and Development, London School of Hygiene and Tropical Medicine, London, UK

**Keywords:** Young People Living with HIV, Antiretroviral therapy, Adherence, Non-adherence, Experiences, Perceptions, Sub-saharan Africa

## Abstract

**Background:**

Young people in sub-Saharan Africa (SSA) shoulder a disproportionate burden of global HIV. We conducted a scoping review to map the research on the experiences and perceptions of young people living with HIV (YPLHIV) on antiretroviral therapy (ART) in SSA to inform future research.

**Methods:**

Following scoping review guidelines, we searched PubMed, Web of Science, EBSCOhost including Academic Search Complete, APA PsycInfo, Health Source: Nursing/Academic Edition, Medline with Full-text, Scopus and ScienceDirect for papers on YPLHIV adhering and not adhering to ART in SSA. We included literature published between 1 January 2010 and 30 September 2022. Search terms employed were adherence, non-adherence, and related synonyms. Bibliometric data and themes describing factors influencing the experiences and perceptions of ART adherence and non-adherence were extracted.

**Results:**

Of the 2671 papers identified, 22 papers from 12 countries were included. Studies employed quantitative (3), mixed (6), and qualitative (13) methods. Most publications concentrated on barriers to adherence rather than enablers. Factors affecting ART adherence and non-adherence were psychosocial, emotional, self-management, support, financial and structural. YPLHIV also faced problems with the responsiveness of health services and access to information.

**Conclusions:**

We identified multiple factors surrounding ART adherence and non-adherence impacting the health and wellbeing of YPLHIV. The review findings showed the importance of research to improve the understanding of the relationships that YPLHIV in SSA develop with ART in adolescence and factors that facilitate adherence. Psychosocial adherence support and patient-centred care approaches are required.

**Supplementary Information:**

The online version contains supplementary material available at 10.1186/s12889-025-22579-6.

## Background

Despite improvements in antiretroviral therapy (ART) uptake through changes in eligibility criteria and medication advancements over the past decades [1–3], adolescents in sub-Saharan Africa (SSA) shoulder a disproportionate burden of the global HIV epidemic. In 2023, 89% of the estimated 1.47 million adolescents and/or young people (YP), living with HIV, aged 10–19 years worldwide were in SSA [4, 5]. Young people living with HIV (YPLHIV) have comparatively poorer treatment outcomes than adults [6–9], and in SSA, the rates of mortality and loss to follow-up (LTFU) among YP living with vertically acquired HIV surpass those in other regions [10, 11]. This highlights the urgent need for interventions to adherence support for YPLHIV in SSA.

There is an increasing number of children living with HIV surviving into adolescence [11–13], and YPLHIV have struggled with achieving optimal adherence [4], particularly in SSA [14–16], presenting challenges to achieving the UNAIDS 95 - 95- 95 targets [17–19]. There is a growing emphasis on providing adherence support to YPLHIV to minimise the risk of disengagement from HIV treatment and care [20]. For YPLHIV taking ART every day, the physical, emotional, behavioural, cognitive and psychosocial experiences of transitioning into adulthood, may exacerbate treatment challenges [21–24], and affect their wellbeing [25–28].

Barriers to ART adherence, include medication-related (e.g. side-effects, regimen changes, fatigue, and treatment options); psychosocial and emotional (e.g. stigma), lack of support (e.g. family, community and peers), and structural factors (e.g. food instability and socio-economic) [8, 9, 29–31]. ART adherence is also influenced by health system and service factors including the availability of youth-friendly health facilities and support services; service delivery and differentiation of HIV care; education information and communication; and the provider-patient relationship [9, 21, 29, 32–34]. These multifaceted barriers can further increase the risk of non-adherence and the likelihood of unsuppressed viral load, poor retention in care, treatment failure, declining health, and HIV transmission [6, 8, 10, 22].

The published literature on the perspectives of YPLHIV has shown the complex nature of treatment taking and ART adherence [35, 36], and a systematic review and Delphi analysis on YPLHIV on ART in SSA, showed varying needs from YPLHIV as patients receiving HIV treatment and care, caregivers, and healthcare providers [37]. This suggested that the experiences and perceptions of YPLHIV within the context of taking or not taking treatment may not be fully understood [38]. We conducted a scoping review to map the existing evidence on factors influencing ART adherence and non-adherence among YPLHIV.

## Research question

This scoping review was guided by the research question: “What experiences and perceptions of YPLHIV in HIV treatment and care in SSA have influenced adherence and non-adherence to oral ART?”

## Methods

### Design

Our scoping review approach was guided by the methodology from Arksey and O’Malley in 2005 [39]. We used their five steps to guide the rigour, reliability, and replicability in this review: we (1) identified the research questions, (2) developed the search strategy to identify the evidence, (3) selected the evidence, (4) extracted and charted the data, and (5) collated, summarised, and reported the findings. We followed the Preferred Reporting Items for Systematic reviews and Meta-Analyses extension for Scoping Reviews (PRISMA-ScR) guideline [40], to ensure that our scoping review adheres to the reporting standard (See the Supplementary File 1).

### Search strategy

In September 2022, we searched the databases: PubMed, Web of Science, EBSCOhost including Academic Search Complete, APA PsycInfo, Health Source: Nursing/Academic Edition, Medline with Full-text, Scopus and ScienceDirect for potentially eligible papers. The MeSH terms were selected using PubMed (information on search strings is available in the Supplementary File 2).

### Eligibility criteria

The population–concept–context (PCC) framework guided the identification of papers according to the inclusion and exclusion criteria [41] (See the Supplementary File 3).

The population is “adolescent” or “youth” or “teenager.” The World Health Organization (WHO) definition of YP was used and included the younger “adolescents” (10–19 years) and older “youth” (15–24 years) [42]. Literature on children below 10 years only and adults above 24 years only was excluded. This was determined using participant range, mean or median age.

The population of focus was YPLHIV accessing or on HIV treatment or enrolled for ART services. This included both HIV and HIV co-morbid conditions as factors surrounding treatment adherence appear to be similar [30]. Literature on YP taking medicines for other conditions was excluded.

The concepts were ART or combination antiretroviral therapy and adherence. The medication was ART to treat HIV also known as antiretrovirals (ARVs). The terms “antiretroviral” and “antiretroviral therapy” were included in the PubMed MeSH terms. Synonyms were included to search widely for research on adherence and non-adherence (See the Supplementary File 3). Literature on Treatment as Prevention (TasP), ARVS for pre-exposure prophylaxis (PrEP), post-exposure prophylaxis (PEP) and ARV-based microbicides were excluded. This was because the scoping review focused on the HIV context of YPLHIV already in care rather than preventing HIV acquisition. To provide a comprehensive review of the existing literature both adherence and non-adherence concepts were incorporated. For this scoping review adherence was defined as taking ART and non-adherence as ever missing a dose.

The context was sub-Saharan Africa. This region selected due to the significant global impact of HIV on YPLHIV with adherence patterns being notably more suboptimal compared to those in developed countries [4, 8, 10, 11]. This included the names of the different countries in sub-Saharan Africa.

### Study selection

We included research published between 1 January 2010 and 30 September 2022. The rationale for the period was based on the WHO publication of the (2010) ART guidelines, for earlier initiation of ART in adults and adolescents with a CD4 count of ≤ 350 cells/mm3 [1], thereby increasing the ART uptake in YPLHIV.

Research articles reporting data on perceptions and experiences of YP adhering and non-adhering to ART were included. Publications focused only on measuring adherence and assessing associations between adherence variables were excluded. Articles investigating treatment outcomes, and retention and loss-to-follow-up were excluded.

### Screening, data charting and synthesis

**Fig. 1:**
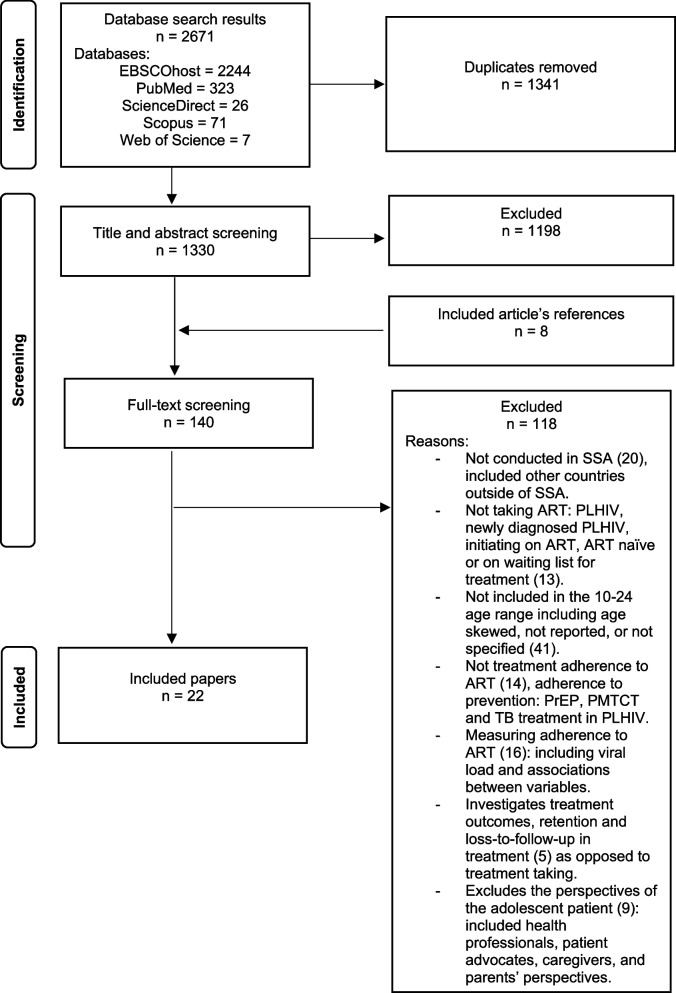
PRISMA flow diagram of selection process

The datasets from the databases were imported into Rayyan, a review software for merging datasets and screening. After the first author (TCS) removed duplicates by screening the merged dataset using Rayyan, the remaining (1330) titles and abstracts were screened for eligibility and study selection. Please see Fig. 1 for the PRISMA flow diagram for papers retrieved, screened, and included. The data extraction was piloted on a subset of studies, refined, and subsequently performed by TCS. The authors TCS and NN then screened the 140 papers for eligibility and JS validated the screened papers. TCS extracted bibliometric characteristics on author, title, year of publication, country, participants and sample size (n), study design, mode of acquisition, age, duration on ART and adherence into a table (Table 1).

**Table 1:** Characteristics of the included papers

**Author, year of publication**	**Country**	**Participants and sample size**	**Study design**	**Mode of acquisition**	**Age range/mean (Category)**	**Duration on ART**	**Adherence and/or non-adherence**
Ankrah et al., 2016 [43]	Ghana	Adolescents living with HIV (19)	Mixed methods	Vertical and horizontal	12–19 years (Adolescent and youth)	≥ 6 months	Adherence and non-adherence
Burns et al., 2020 [44]	Malawi	Adolescents living with HIV (16), Caregivers (16) and Community members (7)	Qualitative cross-sectional	Unknown/assumed	10–19 years (Adolescent and youth)	≥ 6 months	Adherence and non-adherence
Chory et al., 2022 [45]	Kenya	Adolescents living with HIV (29)	Mixed methods	NR	9–19 years (Adolescent and youth)	NR	Adherence
Cluver et al. 2015 [46]	South Africa	Adolescents living with HIV (684)	Mixed methods	Vertical and horizontal acquired	10–19 years (Adolescent and youth)	≥ 6 months	Adherence and non-adherence
Denison et al., 2015 [47]	Zambia	Adolescents living with HIV (32) and Caregivers (23)	Qualitative cross-sectional	Unknown/assumed	15–18 years (Adolescent and youth)	NR	Adherence and non-adherence
Enimil et al., 2016 [48]	Ghana	Adolescents living with HIV (40)	Mixed methods	Vertical	12–19 years (Adolescent and youth)	NR	Adherence
Fetzer et al., 2011 [49]	Democratic Republic of the Congo	Children living with HIV (18) and Caregivers (18)	Qualitative cross-sectional	Unknown/assumed	8–17 years (Adolescent and youth)	≥ 6 months	Adherence
Gichane et al., 2018 [50]	Tanzania	Adolescent orphans living with HIV (17)	Mixed methods	Vertical	13–24 years (Adolescent and youth)	NR	Adherence
Gordan et al., 2022 [51]	Uganda	Adolescents living with HIV (249)	Mixed methods	NR	10–19 years (Adolescent and youth)	≥ 6 months	Adherence
Horter et al., 2019 [52]	Eswatini	Asymptomatic people living with HIV (30)	Qualitative longitudinal	NR	17–25 years (Youth) and 26–49 years (Adult)	NR	Adherence
Kawuma et al., 2014 [53]	Uganda	Children living with HIV (26)	Qualitative longitudinal	NR	11–13 years (Adolescent)	NR	Non-adherence
Kim et al., 2017 [54]	Malawi	Adolescents living with HIV (562)	Cross-sectional survey	Vertical	12–18 years (Adolescent and youth)	≥ 72 months	Non-adherence
Kunapareddy et al., 2014 [55]	Kenya	Adolescents living with HIV (23)	Qualitative cross-sectional	Vertical	10–16 years (Adolescent and youth)	> 12 months	Adherence
Li et al., 2010 [56]	South Africa	Young people living with HIV (26)	Qualitative cross-sectional	Vertical and horizontal	7–15 years (Adolescent)	NR	Adherence
Loades et al., 2018 [57]	South Africa	Adolescents living with HIV (14)	Qualitative cross-sectional	NR	11–18 years (Adolescent)	NR	Non-adherence
MacCarthy et al., 2018 [58]	Uganda	Adolescents living with HIV (25), Healthcare Providers (15) and Community members (9)	Qualitative cross-sectional	Unknown/assumed	14–24 years (Adolescent and youth)	> 3 months	Adherence and non-adherence
Madiba and Josiah, 2019 [59]	Botswana	Adolescents living with HIV (30)	Qualitative cross-sectional	Vertical	12–19 years (Adolescent)	> 12 months	Adherence and non-adherence
Matovu et al., 2012 [60]	Uganda	Young women living with HIV/AIDS (6)	Qualitative cross-sectional	NR	15–24 years (Youth)	> 12 months	Adherence
Mutwa et al., 2013 [61]	Rwanda	Adolescents living with HIV (42) and Caregivers (10)	Qualitative cross-sectional	Vertical	12–21 years (Adolescent and youth)	≥ 36 months	Adherence
Sherr et al., 2018 [62]	South Africa	Adolescents living with HIV (1058)	Cross-sectional survey	Vertical and horizontal	13.76 years (Adolescent)	NR	Adherence
Tarantino et al., 2022 [63]	Ghana	Young people living with HIV (32)	Qualitative cross-sectional	Vertical and horizontal	18–24 years (Youth)	NR	Adherence
Teasdale et al., 2021 [64]	Mozambique	Adolescents living with HIV (208)	Cross-sectional survey	Vertical and horizontal	15–19 years (Adolescent and youth)	NR	Adherence

*ART* Antiretroviral therapy, *NR* Not reported

We used a stepwise approach for data analysis. Initially, TCS collated the findings related to themes. We interpreted the themes adapting an approach of Chem and colleagues in 2022 [38], using the perspective of the needs of YPLHIV on ART. We applied this to the findings and analysed the papers for identification and frequency of themes. We constructed Table 1 to show the bibliometric characteristics of included papers and Table 2 summarising the experiences and perceptions of YPs adherence to ART. This provided a comprehensive visualisation of the needs-based factors influencing the young person’s relationship with ART in terms of adherence and non-adherence.

**Table 2:** Experiences and perceptions of YPLHIV in HIV treatment and care adhering and not adhering to ART

**Type of factor**	**Experience and perception themes**	**Influence on taking ART**
Psychosocial and emotional	Perceived and experienced stigma [43–45, 48–52, 54–56, 58–62, 64]	Proximal
	HIV disclosure difficulties [44, 46–49, 53, 55, 58, 59, 61, 64]	Proximal
	Secrecy during taking ART [44, 48, 50–53, 55, 56, 58, 59, 61, 64]	Proximal
	Acceptance of HIV diagnosis [52, 61]	Proximal
	Motivation through health, knowledge, and beliefs [43, 45, 48, 49, 52, 56, 61]	Proximal
Self-management and medication adherence	Forgetting to take prescribed ART [43–45, 47–51, 54, 55, 58–61, 63]	Proximal
	Treatment fatigue [48, 49, 55–58, 60]	Proximal
	Challenges in tolerating ART regimens [43, 48, 51–55, 57, 58, 60, 61]	Proximal
Family and community support during HIV treatment and care	Need for family support [43, 44, 47–51, 53, 55, 56, 58–61, 63]	Proximal
	Communication challenges, violence, abuse, and neglect [44, 50, 53–56]	Proximal
	Inadequate community and social support [44, 45, 47, 51, 53, 56–58, 62]	Proximal
	Lack of adolescent autonomy during treatment [47, 60]	Proximal
	Self-care and responsibilities for others [60, 61]	Proximal
Financial and structural	Food insecurity [43, 48, 49, 51, 53, 56, 58, 59, 61, 63, 64]	Distal
	Limited access to drinking water [43, 48, 51, 63, 64]	Distal
	Transport costs [43, 59, 63]	Distal
Responsive health system and services	Lack of adolescent and youth-friendly HIV services [44, 46, 48, 49, 51, 55, 56, 62, 64]	Distal
	Need for healthcare provider support [43, 44, 48, 49, 54, 57, 62, 63]	Distal
	Mental health interventions for HIV-related depression and stress [45, 48, 51, 53, 54, 57, 62]	Distal
	School system not considerate of YPLHIV needs [48, 58, 59, 61]	Distal
	Negative health provider attitudes towards YPLHIV when they miss ART [51, 53, 55]	Distal
	High facility turnaround time during ART refills [44]	Distal
Normalcy and aspirations	Desire to socialise with peers [43, 48, 53]	Proximal
	Desire for a normal and healthy life or future [43, 48, 51, 52, 56, 59, 61]	Proximal
	Desire to love and be loved in return [48, 51, 60]	Proximal
Information about HIV and ART adherence	Misinformation, misconception, and misperceptions [47, 49, 52, 55–57]	Proximal
	Desire for more knowledge [43, 48, 57, 64]	Proximal

*ART* Antiretroviral therapy, *YPLHIV* Young People Living with HIV

The findings were presented using a narrative approach guided by themes identified in the papers. These were synthesised into narratives by integrating data from Tables 1 and 2. The themes were categorised using Bronfenbrenner’s Ecological Systems Theory into proximal and distal themes [65]. In the context of ART adherence and non-adherence, proximal themes refer to factors directly related to YPLHIV taking or not taking medication (oral ART) and distal factors were those which indirectly influenced YPLHIV taking or not taking medication (oral ART) [65].

## Results

### Characteristics of included studies

The search yielded a total of 2671 identified titles from the databases. After removing duplicates and irrelevant texts, 1330 relevant titles were screened by abstract. The 1198 abstracts that did not fit the inclusion and exclusion criteria were excluded. An additional 8 articles were identified from reference lists of articles as potentially eligible, contributing to a total of 140 that were included for full-text screening. Papers focusing only on adherence measures (16 papers), health outcomes (five), and non-adolescent perspectives (nine) were then removed. The screening resulted in a total of 22 papers for data analysis (see Fig. 1).

The 22 primary research studies in SSA were focused on 12 countries: South Africa (*n=*4), Uganda (*n=*4), Ghana (*n=*3), Kenya (*n=*2), Malawi (*n=*2) Botswana (*n=*1), Democratic Republic of the Congo (*n=*1), Eswatini (*n=*1), Mozambique (*n=*1), Rwanda (*n=*1), Tanzania (*n=*1), and Zambia (*n=*1) (Table 1). The majority of the 22 papers were published between 2012 and 2019.

Of the 22 research papers, 13 reported studies that used qualitative methods, four used mixed methods and three used quantitative methods employing cross-sectional survey designs (Table 1). Of the 13 qualitative studies, eleven used cross-sectional research designs [44, 47, 49, 55–61, 63], and two analysed retrospective data collected longitudinally [52, 53].

Thirteen papers focused on factors influencing adherence [45, 48, 49, 51, 52, 55, 56, 59–64]. Six papers focused on both adherence and non-adherence [43, 44, 46, 50, 58, 59] and three on non-adherence [53, 54, 57] (Table 1).

Four papers included the perspectives of caregivers' [44, 47, 49, 61], of which one included community members' perspectives [44]. One paper included community members' and healthcare providers' perspectives [58]. ART was orally self-administered treatment, in the form of tablets and syrups while medicines for co-morbid conditions were not reported.

### YPLHIV on ART

Fourteen papers reported on participants covering both adolescent (10–19 years) and youth (15–24 years) age groups [43–51, 54, 55, 58, 61, 64], five on adolescents [53, 56, 57, 59, 62], and three on youth of which one included representation from adults [45, 49, 56] (Table 1).

Six papers reported the factors from YP with vertically acquired HIV. Four of these papers reported on YPLHIV on ART for more than 12 months and had acquired HIV vertically [54, 55, 59, 61]. Six papers reported from both vertically and horizontally acquired YPLHIV [43, 46, 56, 62–64]. Six papers did not specify the mode of acquisition [45, 51–53, 57, 60], and in four papers this was unknown or assumed [44, 47, 49, 58] (Table 1).

### Treatment-taking influences on YPLHIV

All the papers reported barriers to care for YPLHIV [43–61, 63, 64], and entailed aspects in the life of a young person on ART, that had both distal and proximal influences on taking ART which were reflected in the themes (Table 2).

Conversely, positive enablers facilitating ART adherence were less common and were proximal to taking treatment (Table 2). Several authors reported that motivation of YPLHIV, to adhere to ART is influenced by factors such as observing health improvements, accepting their HIV status, possessing accurate knowledge about HIV, and having religious faith [43, 45, 47–49, 52, 55–57, 61, 64].

Certain experiences and perceptions were both barriers and enablers and proximal to taking and adhering to ART. The need for family and community support, desire for a normal and healthy life or future, to socialise with peers and experience love and belonging were themes found to both hinder and facilitate treatment-taking [43–45, 47–51, 53, 55–63]. These social relationships provide support. However, they also encourage YPLHIV to be cautious or secretive when taking ART due to fears of their HIV status being discovered.

### Health and wellbeing of YPLHIV on treatment

Multiple factors affecting the health and wellbeing of YPLHIV on ART in SSA were described in the papers (Table 2).

### Psychosocial and emotional factors

*Perceived and experienced stigma* associated with the HIV status of YPLHIV was prevalent and had various consequences. This stigma was reported in papers by Kim and colleagues in 2017 and Teasdale and colleagues in 2021) [54, 64]. Stigma resulted in fear of unintended disclosure and led to the YPLHIV avoiding clinic appointments for refills and being reluctant to disclose their status [59]. YPLHIV faced teasing from peers and community members about their ART use [49], and rumours about their status after being seen taking their treatment [58]. Researchers found that some caregivers to YP who acquired HIV vertically, also experienced stigma which led them to discard the young person’s ART in attempt to prevent unintended disclosure [50]. A study in Eswatini found that although YP were motivated to initiate ART early to protect their health, they feared being stigmatised by engaging in treatment and care [52]. Some hid their ART, missing doses when in the presence of other people, and others stopped attending clinics for ART refills in fear of being seen.

*HIV disclosure difficulties* included incomplete or non-disclosure by parents and caregivers resulting in the lack of understanding among both adolescent and youth groups regarding why they needed to take ART [49, 53, 56, 58]. The lack of consistent HIV status knowledge impacted ART adherence for YPLHIV aged 10–19 years in South Africa [46]. In Uganda, incomplete disclosure led to suspicion about what the ART was for and contributed to non-adherence in younger adolescents aged between 11 and 13 years [53]. In Zambia, caregivers of adolescents and youth 18 years and under in, expressed concerns about unintended disclosure and preferred to keep the young person's HIV status confidential within the household [47, 53]. In Uganda and Botswana students, aged 12–24 years who were orphaned and in boarding school, missed their ART doses because they were reluctant to collect treatment from clinics and take their drugs in communal spaces fearing accidental disclosure from the noise of ART in pill containers [58, 59]. While research in Malawi found that a few YP (15–19 years) shared their HIV status [64], other studies found that YPLHIV in adolescent and youth age groups chose not to disclose their HIV status to friends, teachers, family members, and partners and this secrecy posed challenges to adherence to ART [51, 56, 58, 59]. For these age groups in South Africa, positive disclosure experiences were associated with better adherence while fear-inducing disclosure strategies at home or in clinic settings were linked to the risk of rebellion through non-adherence [46].

*Acceptance of HIV diagnosis* varied, ranging from low acceptance, marked by anger, demotivation, and reluctance to take ART, to high acceptance, marked by a willingness to live and overcome stigma [61]. Research conducted in Eswatini and Rwanda revealed that some YP did reach acceptance and needed time to process and come to terms with their HIV status [52, 61]. Difficulties in accepting their HIV diagnosis were linked to feelings of depression, isolation and increased risks of ART non-adherence. For others it led to feelings of anger and confusion about why they had acquired HIV and their siblings had not [61]. Conversely, some YPLHIV in Eswatini who had accepted their status perceived no difference between themselves and others, displaying an absence of self-stigma as a barrier to ART adherence [52].

*The secrecy during YPLHIV their taking ART* was evident in many papers [44, 48, 50–53, 55, 56, 58, 59, 61]. Some YPLHIV were reported to store their ART discreetly [43, 55], and younger adolescents chose not to take it when playing with friends or when others were around them at home [53]. This limited their reminders to take their ART to those family members who were aware that they were taking ART or their HIV status and were available to assist with this. For these YP, under 13 years, this may be exacerbated by these caregivers not having the time and availability to give them their ART at the prescribed time [53]. Earlier research in the Democratic Republic of the Congo revealed that caregivers were afraid that YPLHIV who knew their HIV status might accidentally disclose it to friends, friends' parents, and community members, leading to stigma [49].

Taking ART was also influenced by *motivation through health, knowledge, and beliefs.* In Ghana, Ankrah et al., reported that YPLHIV gain motivation through focusing on good health while taking ART, along with positive perceptions of the potential of ART to support a long life, knowledge about HIV and beliefs of ART being made by God [43]. In Eswatini, YPLHIV were encouraged to stay on ART by observing the poor health of others in their community who had started treatment later in their journey with HIV [52]. In Kenya, YPLHIV who received support through an online intervention reported that they believed in God and had hope for the future [45]. Some healthcare providers noted that taking ART could expose Christian YPLHIV to judgment from their community due to moral and religious values, such as abstaining from sexual intercourse before marriage [52]. These YP were assumed to have acquired HIV through sexual transmission, which carried a stigma leading to feelings of shame, humiliation, and even suicidal ideation [52]. In Zambia, while some YPLHIV were deterred from adhering to ART by their hope for divine healing, others in Zambia and Rwanda continued with their treatment despite holding these beliefs [47, 61].

### Self-management and medication adherence factors

Adherence challenges varied among individuals, highlighting the need for tailored support. Some YPLHIV used watches, clocks, or phone alarms as reminders to take their medication [47]. It was reported that YP were *forgetting to take their prescribed ART* on time because of life events, for example, a young male had forgotten to take his treatment on time because of going out to socialise with peers [43]. There was a reluctance to disturb social events to take treatment and a hesitancy to take their ART when with peers to avoid unintended disclosure [47]. It was reported that YP experienced *treatment fatigue* [47–49, 55–57, 60], from the daily burden of taking ART and appeared to have difficulty in identifying and developing coping strategies. The extent of this fatigue was reported in a study conducted in Uganda where pill burden and commitment to ART led to breaks in treatment or drug holidays [58].

The taste, size, number, and frequency of pills, taking of dispensed formulation of ART and treatment side effects all influenced ART adherence [43, 55]. *Challenges in tolerating ART regimens* included tablets being too large to swallow, often getting stuck in the throat of YP, and syrups having a bitter taste [55]. In Kenya, the number of tablets and the measuring of syrups in a spoon were also reported as medication user-related challenges when consuming ART [55]. In Ghana there was reported to be a preference for tablets because of their portability [43]. Challenges in tolerating ART regimens were apparent in research conducted in Botswana and the Democratic Republic of the Congo, and it was reported that YPLHIV experienced treatment side effects. These included dizziness, vomiting, nausea, loss of taste, sleep disturbances, light-headedness; and a burning sensation in the stomach, all said to be caused by taking ART on an empty stomach [49, 59]. Food insecurity, a financial and structural factor, posed significant barriers to self-management and medication adherence [48, 51, 58, 63].

### Family and community support during HIV treatment and care factors

There was *a need for family support* to help YPLHIV cope with the condition across adolescent and youth groups [43, 44, 47–51, 53, 55, 56, 58–61, 63]. This encompassed various aspects such as monitoring, reminders from those disclosed to, motivational and emotional assistance and accompaniment to the clinic [50, 51, 53, 63], and guidance on whether to take ART on an empty stomach [53, 59]. This support for YPLHIV on ART extended to peers [48], and the community. For example, there was a neighbour who provided food to help YP between 11 and 13 years taken ART who had disclosed that a lack of food was a reason for missed doses [53]. Studies from Malawi and Uganda have shown that some YPLHIV did not receive the necessary support from their caregivers [44, 51, 53]. Instead, some YP experienced *communication challenges, violence, abuse, and neglect* from their caregivers [44, 53–55]. Kunapareddy et al. in research in Kenya showed YPLHIV’s negative interactions with caregivers, describing these as authoritative, abusive, and argumentative [55]. Along with research conducted in South Africa, it was reported that ART non-adherence among YPLHIV was driven by rebellion in response to negative interactions with guardians [53, 56]. Some YP held negative perceptions of their caregivers from the lack of support they received [44]. Research in Uganda found that the lack of support from caregivers and partners appeared to result in negative emotions and suicidal thoughts [53, 60], and was associated with non-adherence. It was reported that some YPLHIV who were non-adherent, were reprimanded by family members and others were abused [44, 53, 55]. For example, some YP were beaten by a grandmother for discarding their ART and emotionally abused by extended family members and guardians [44, 53, 55]. In research in Kenya these abusive experiences from guardians appeared to have a negative effect causing discomfort in YP and discouraging adherence [55]. The interference with the ART adherence of orphans and their neglect was evident among caregivers in research conducted in Tanzania [50]. The relationships included stepmother, sister and grandmother, where challenges ranged from preventing medication storage in the refrigerator, throwing ART out, withholding food to take before treatment and not providing funds for transport to collect ART at health facilities [50].

There was a lack of caregiver encouragement to take ART and be part of the young person’s treatment plan as demonstrated in a study in Uganda [53]. It was reported that before belonging to a mobile support group, YP felt that there was no one else with an “HIV positive status”, indicating the importance of a sense of community to the wellbeing of YPLHIV on ART [45]. Some research findings point to *inadequate community and social support* [44, 45, 47, 51, 53, 56–58, 62], and included examples of peer exclusions and community strategies toward YPLHIV aged 11–18 years [57]. In Malawi, YPLHIV who were non-adherent faced discipline from community members in the form of fines and prohibitions on recreational activities [44]. This approach was reported as being synonymous with that of the threatening and monitoring nature reflected in the healthcare professionals providing HIV treatment and care services to YPLHIV [44]. Research conducted in Zambia, South Africa and Uganda reported a *lack of adolescent autonomy during treatment* in decision-making for treatment based on the nature of their relationship with the caregiver [47, 56, 60]. For example, whilst some caregivers transitioned to YP assuming the responsibility of taking their ART independently as they aged, others kept it with them and administered it to them, whilst adherence for some YP was facilitated by their respect and admiration toward caregivers [47, 60]. YPLHIV experienced a lack of self-care and responsibilities for others, and this was reported in two papers conducted in Uganda where YPLHIV had to provide an income in a child-headed household and carried the duty of cleaning the home [58, 60]. It appeared that these responsibilities negatively influenced treatment taking for YPLHIV on ART.

### Financial and structural factors

*Food insecurity* posed a significant obstacle to YPLHIV in adhering to ART for both adolescents and youth [43, 48, 49, 51, 53, 56, 58, 59, 61, 63, 64], with a more pronounced impact observed in Uganda and Ghana [43, 48, 51, 53, 58, 63]. Child-headed households in particular those where YPLHIV 14–24 years who had been on ART for less than three months, struggled because of unstable incomes that hindered their ability to afford food and had caused them stress [58]. In Uganda and Botswana [53, 59]. YPLHIV often missed their ART doses when there was no food to eat. Some youth chose not to take their ART because of this whilst caregivers of children in adolescent and youth groups discouraged taking ART to protect them from the discomfort and “side effects” of taking medication on an empty stomach [44, 49, 53, 59]. This suggests that the theme of challenges in tolerating ART regimens is more complex in some countries in SSA. A study in Malawi showed that YPLHIV had negative perceptions of their caregivers who as disciplinarians made it compulsory to take ART without eating food [44], further complicating treatment adherence for YPLHIV taking ART.

In research conducted in Ghana and Rwanda authors reported *limited access to drinking water* was a barrier to consuming oral ART [43, 48, 61]. The transport costs were expenses associated with travelling long distances to attend clinic appointments for HIV care delayed YP, compromising their treatment and overall health [43, 48, 63].

### Responsive health system and services factors

YP on ART in several countries, including Malawi, South Africa, Ghana, Democratic Republic of the Congo, Uganda, Kenya, and Mozambique experienced issues related to *a lack of adolescent and youth-friendly HIV services* [44, 46, 48, 49, 51, 55, 56, 62, 64]. The existing interventions in health facilities failed to address the specific drivers of non-adherence among YP and did not consider their circumstances, vulnerabilities, and the overall wellbeing of YPLHIV concerning treatment adherence [44, 62, 64]. A study in Malawi reported that pill counts contributed to *long facility turnaround times during ART refills* [44]. YP perceived that the pill counts, longer facility waiting times and more frequent appointments helped enforce discipline when health professionals’ expectations for adherence were not met. Although there appeared to be no intention to lengthen ART dispensation, waiting times and appointment frequency, this perception contradicted the concept of adolescent-friendly services associated with improved adherence.

In Kenya and Uganda, there were negative health provider attitudes towards YPLHIV when they missed ART doses [51]. YP (between 11 and 13 years) did not communicate their missed ART doses to healthcare providers, for fear of disappointing them, whilst some alluded to their fear of healthcare providers based on previous experiences of verbal abuse [53]. Their caregivers were reported to follow suit, in ineffective communication about missed doses and felt responsible for their children's non-adherence [53]. The need for healthcare provider support included non-adherence treatment support, medication assistance and responding to psychosocial and emotional needs including providing mobile HIV support to reduce the burden of travel to the clinic [48, 49, 63].

Mental health services were found to be essential for YPLHIV on ART in Kenya, Ghana, Uganda, Malawi, and South Africa [45, 48, 51, 53, 54, 57, 62]. YP experienced HIV-induced depression, anxiety, stress, suicidal ideation, and substance abuse [54, 62]. It appeared that there were unmet mental health interventions for HIV-related depression and stress. Additionally, YPLHIV on ART withdrew from social activities with peers due to fatigue, underscoring the importance of psychosocial support [57]. Research in South Africa highlighted the presence of mental health challenges, suicidality, and substance abuse among YPLHIV acquired horizontally rather than vertically, suggesting the need for tailored support based on variations in experiences in YP with an average age of 14 years [62].

Authors of papers on research conducted in Ghana, Uganda, Botswana, and Rwanda reported the school system as not being considerate of YPHIV needs. The school schedule often conflicted with the timing of ART doses, making it difficult for YPLHIV to adhere to ART during school activities including trips, sports and examinations. There appeared to be limited support in schools to help YP manage their treatment [48, 58, 59, 61]. It appeared that the system was not organised for YP in HIV treatment and care. Moreover, the revision of schedules was further challenged by the unwillingness of YPLHIV aged 12–19 years to disclose their HIV status to the teachers [59].

### Normalcy and aspirations factors

Socialising with peers served as both a distraction and a source of support for YPLHIV. In Uganda for younger adolescents, there was a prioritisation of spending time on social activities with peers, diverting their attention from adhering to ART [53]. YPLHIV had a desire to socialise with peers, engage in recreational activities and maintain playtime with peers [43, 48, 53]. It appeared that socialising required them to often be in public spaces away from their homes where their medication was usually stored and accessed, which led to treatment interruptions.

For some YPLHIV, staying on ART provided hope and the possibility of living a normal life, improved physical health and enhanced appreciation for managing HIV [43, 48, 51, 52, 56, 59, 61]. The desire for a normal and healthy life or future appeared to facilitate taking ART. A study in Rwanda reported that YLHIV wanted to live as their “uninfected peers” were and in terms of the clinical experience hoped for an ART regimen that involved taking ART less frequently [61]. In a similar light, research in Botswana linked normalcy to the lives of YP living with a negative HIV status [59, 61]. YPLHIV (aged 10–24 years) had a desire to love and be loved in return. In Ghana, research showed the importance of plutonic love and YPLHIV forming friendships with peers who could empathise with their experiences and provide hope and motivation through peer support [48]. Some YP had close had close friends who provided a space for sharing, which fostered a sense of feeling loved and belonging [51]. Women (15–24 years) living with HIV in Uganda in relationships expressed a desire for romantic love, for fertility and marriage despite perceiving these goals as challenging due to their HIV status and having concerns about medication side effects [60]. They reported experiencing stress and fear of stigma around partners and friends leading to secrecy during taking ART and the choice to withhold information on their HIV status.

### Information about HIV and ART adherence factors

Some YPLHIV had inaccurate information about ART being a cure [55]. Among YPLHIV on ART, there were misinformation, misconceptions, and misperceptions about HIV and ART adherence [47–49, 52, 55, 57]**.** Some believed that ART was manufactured from body parts of the deceased. Some explained that ART by preventing the visible symptoms of HIV prevented a person distinguishing between healthy and sick sexual partners, increasing the risk of unprotected sex and potential HIV transmission [55]. In Kenya, YPLHIV were reported to use traditional, alternative, or complementary therapies [55]. There were misperceptions about fatigue and its impact on daily tasks and social activities with peers at school. YPLHIV felt distressed when they fell asleep at school, reprimanded by teachers and felt isolated when they were unable to participate in social activities with friends [57]. These individuals 18 years and under did not understand that their fatigue was related to being on ART [57].

These YP expressed a desire for more knowledge about HIV and ART adherence [43, 56, 57, 64]. For instance, a study in South Africa revealed that YP wanted more information on HIV-related topics in schools and clinics. Understanding the biology of HIV seemed to underscore the importance of adhering to ART [56]. In addition to the concept of treatment fatigue, YP had a lack of understanding of how they acquired HIV and the necessity of taking ART [48, 57]. Some YPLHIV did not understand the link between adherence to ART and their health status [49, 52]. In the Democratic Republic of the Congo, YPLHIV whose HIV status was undisclosed showed a limited understanding of the purpose of adherence [49]. This created a barrier for caregivers to explain the importance of adhering to ART for fear of unintentional disclosure. There was a desire for more knowledge about HIV and ART adherence [43, 56, 57, 64]. For instance, a study in South Africa revealed that adolescents (7–15 years) wanted more information on HIV-related topics in schools and clinics and knowledge about the biology of HIV which appeared to facilitate the importance of adhering to ART [56].

## Discussion

### Summary of main results and strengths

This scoping review looked at research on YPLHIV in the HIV care continuum in SSA identifying their experiences and perceptions on adhering and not adhering to ART. It contributes to the existing research by providing insight into the experiences of YPLHIV who have been on ART for three months or longer in this region, reflecting on persistent barriers to ART adherence from 2010 to 2022. Our review highlights the need to develop adherence support interventions tailored to address the barriers faced by YP and identifies several research gaps. These gaps contribute to the growing body of research on YPLHIV on ART in the HIV care continuum in SSA.

### Gaps identified in this review

The gaps from our review encompassed: understanding the wellbeing of YPLHIV on ART and developing corresponding interventions, insufficient details on the characteristics of YPLHIV including inadequate reporting of modes of HIV acquisition, the shortfall in reported factors that facilitate ART adherence, the need to investigate non-adherence alongside adherence and the lack of research on non-adherence.

We identified a gap in longitudinal research that investigates the experiences of YPLHIV in the HIV care continuum. This points to the need for and value of comprehensive interventions that adopt a whole-person approach and address the needs of YP across their journey through treatment and care. Fewer studies adopted a multi-perspective approach (including caregivers, community members and healthcare providers), limiting the understanding of the relational and systemic factors that influence YPLHIV taking their ART. More in-depth research is needed to fully understand the complexities of the wellbeing of YPLHIV on ART in countries in SSA by considering their treatment-taking social context.

### Developing adherence support interventions

Suboptimal adherence is a shared challenge for children growing up with chronic conditions and transitioning into adult care [14]. The prevalence of the barriers compared to enablers in our review underscores the importance of adapting HIV treatment and care services to accommodate the challenges faced by population group as they transition [66]. To this end, research has identified the need to focus on adherence support (e.g. family, community peers) proximal to the YPLHIV on ART [15, 36, 67–70], and to expand interventions into social spaces [71, 72], addressing financial and structural barriers proximal to taking ART, and strengthening care models. This points to the value of differentiated care to respond to the specific needs, and wellbeing of YPLHIV on ART.

The review identified a range of multifaceted barriers faced by YPLHIV on ART [43, 44, 53]. These are analogous to findings from previous research involving YP in SSA [8, 9, 29–31], highlighting the vulnerabilities of this population group. The persistent need for support was consistently observed within the household setting, emphasising the need for interventions that address factors within the immediate ecosystem of treatment adherence.

Our review has highlighted the negative experiences of YP growing up with HIV, revealing a suboptimal quality of life [48]. YPLHIV face treatment fatigue, challenges in tolerating ART regimens, difficulties in accepting their HIV diagnosis, disclosure issues, violence, abuse, neglect, and stigma, all affecting their health and wellbeing. Researchers advocate for a holistic patient-centred HIV care approach to address the comprehensive needs of YPLHIV on treatment [73]. Our findings underscore the need to develop and adapt interventions that address the wellbeing of YPLHIV [26]. Further research is needed on wellbeing as it relates to adherence and its role in understanding YPLHIV on ART in SSA [74, 75].

### Characteristics of YPLHIV

The review findings indicate that YPLHIV on ART have diverse identities that are underpinned by challenges, for example: loss of parental care, violence, neglect, abuse, food insecurity, and poverty [43, 44, 46, 48–51, 53–58, 60, 61, 63, 64]. These difficulties impact their treatment and care continuity.

In SSA, access to data is required for designing interventions that support ART adherence and are tailored to meet the needs of all YP [34, 76, 77]. In research YP acquiring HIV through vertical and horizontal modes initiate treatment at different time points in their lives and appear to experience a similar burden taking treatment irrespective of how they acquired HIV [27, 62, 78]. More research is needed to develop an evidence base of these circumstances including information based on the mode of acquisition [44, 45, 49, 51–53, 57, 58, 60].

### Need to investigate non-adherence alongside adherence

Our scoping review findings indicate that various factors within a YPLHIV's social context, contribute to ART non-adherence, including missed doses, treatment interruptions, drug holidays, and non-adherence [43, 44, 46, 47, 58, 59]. To develop evidence-based interventions and provide effective adherence support, a thorough understanding of the reasons for taking and not adhering to ART within this context is critical. There is a clear need and value in investigating both adherence and non-adherence alongside each other, as it offers a more comprehensive understanding of YPs experiences with ART and support with taking ART.

### The importance of non-adherence research

Our review emphasises the importance of understanding the experiences and perceptions of YPLHIV within the HIV care continuum and within the early years of receiving ART when many more barriers than enablers to treatment taking are experienced. In literature, it appears that for some YP their challenges with treatment taking and ART adherence go unnoticed unless they have a negative treatment outcome ‒ are LTFU, not retained in care, have an unsuppressed viral load or treatment failure [10, 14, 79]. Failure to address setbacks and facilitate ART adherence through adherence support interventions for this socially excluded or marginalised population early in care can make later engagement in the HIV care continuum difficult [68, 80–82].

Non-adherence to ART impacts progress towards the 95% viral suppression target for all individuals on ART, including adolescents by 2025 [82, 83], and affects the health and wellbeing of YPLHIV in the HIV care continuum. Addressing adherence and treatment needs in YPLHIV informs the evidence base for and interventions for better HIV care, especially as more people have access to treatment and switched to safer, more effective ART regimens [2, 84].

### Limitations of review

This review has several key limitations. The geographic scope was restricted to SSA, which may limit generalisability of findings to YPLHIV in other regions. Most of the 22 papers were published between 2012 and 2019, potentially missing more recent research. This could be attributed to the interruption or delay of research involving in-person data collection from participants, such as YPLHIV, caused by COVID- 19 social distancing and lockdown measures.

We excluded grey literature and reviews because the aim was not to review literature for programme implementation or policy revision purposes, but to investigate experiences and perceptions of YPLHIV in HIV treatment and care in SSA adhering and not adhering to ART. Information on ART adherence enablers i.e., support and interventions being implemented and revised may have been missed.

We did not include studies involving samples of YP who were not receiving ART. They may not be on ART at the time because they had not yet initiated or stopped the treatment and not returned to care, and challenges related to non-adherence for these YP were not described.

The heterogeneity in study design limited direct comparisons and may have introduced potential bias. This review, however, was guided by a research question that prioritises perspectives of YPLHIV, for which studies including qualitative methods offer deeper insights. There was inconsistent reporting of mode of HIV acquisition, with six papers reporting vertical transmission, and potentially masking experiences related to vertical versus horizontal transmission.

Our application of the WHO categories [42], showed that most papers focused on groups covering both adolescents and youth (14 papers) and fewer studies within each group individually (five in the adolescent group and three in the youth group). Although this framework was useful for reporting, the papers included variations in age group definitions which limited our comparisons and consistency in documenting findings.

## Conclusions

This scoping review mapped research evidence on factors influencing ART adherence among YPLHIV in SSA. These interconnected factors exist both proximal and distal to the taking of ART in the lives of YP, impacting their health and wellbeing. It identified multiple barriers and some enablers across 22 studies from 12 countries. The barriers identified, spanning from 2010 to 2022 demonstrate how adherence difficulties for YPLHIV in SSA have persisted despite advancements in ART, increasing the risk of disengagement in HIV care continuum. We recommend further research that examines the relationship YP have with ART as they transition from adolescence into adulthood and identify further enablers to ART adherence. The findings from the review suggest the need for youth-friendly services and holistic patient-centred care approaches and interventions, that address psychosocial adherence support, provide accurate HIV information, and engage individuals such as family members and peers of the YPLHIV.

## Supplementary Information

Additional file 1. Preferred Reporting Items for Systematic reviews and Meta-Analyses extension for Scoping Reviews (PRISMA-ScR) Checklist.

Additional file 2. Example of search strings used in search strategy.

Additional file 3. Population – concept– context and inclusion and exclusion criteria.

## Data Availability

This published article and its supplementary information files include all data generated or analysed during this study.

## Abbreviations

YPLHIV: Young People Living with HIV
ART: Antiretroviral therapy
SSA: sub-Saharan Africa
HIV: Human immunodeficiency virus
LTFU: Loss to follow-up
UNAIDS: Joint United Nations Program on HIV/AIDS
PCC: Population–concept–context
WHO: World Health Organization
JBI: Joanna Briggs Institute
ARV: Antiretroviral
MeSH: Medical Subject Headings
TasP: Treatment as Prevention
PrEP: Pre-exposure prophylaxis
PEP: Post-exposure prophylaxis
CD4: Clusters of differentiation 4
PLHIV: People Living with HIV
PMTCT: Prevention of Mother to Child Transmission
PRISMA-ScR: Preferred Reporting Items for Systematic reviews and Meta-Analyses extension for Scoping Reviews
