# Supplementary material for: Experiences and perceptions on antiretroviral therapy adherence and non-adherence: a scoping review of young people living with HIV in sub-Saharan Africa
Source: BMC Public Health. 2025 Apr 17;25:1450. doi: 10.1186/s12889-025-22579-6 (PMC12004696; doi:10.1186/s12889-025-22579-6)
Supplement: Supplementary file 2 — Additional file 2. Example of search strings used in search strategy. [file 12889_2025_22579_MOESM2_ESM.pdf]

Additional file 2.

Example of search strings used in search strategy.

1. ((Adolescent OR youth OR teenager OR “young people”) AND;
2. (HAART OR "highly active antiretroviral therapy" OR “combination antiretroviral”) AND;
3. (Adherence OR compliance / persistence / “treatment success” OR non-adherence OR “poor adherence” / “suboptimal adherence” / “treatment interruption”) AND;
4. (“sub-Saharan Africa” OR [Cameroon / “Central African Republic” / Chad OR Congo / “Democratic Republic of the Congo” / “Å‘Equatorial Guinea” / Gabon / “Sao Tome and Principe” / Burundi / Djibouti / Eritrea / Eswatini / Ethiopia / Kenya / Rwanda / Somalia / “South Sudan” / Sudan / Tanzania / Uganda / Angola / Botswana / Lesotho / Malawi / Mozambique / Namibia / “South Africa” / Zambia / Zimbabwe / Benin / “Burkina Faso” / “Cabo Verde” / “Cote d’Ivoire” / “Ivory Coast” / Gambia / Ghana / Guinea / Guinea-Bissau / Liberia / Mali / Mauritania / Niger / Nigeria / Senegal / “Sierra Leone” / Togo])))
