# Supplementary material for: Experiences and perceptions on antiretroviral therapy adherence and non-adherence: a scoping review of young people living with HIV in sub-Saharan Africa
Source: BMC Public Health. 2025 Apr 17;25:1450. doi: 10.1186/s12889-025-22579-6 (PMC12004696; doi:10.1186/s12889-025-22579-6)
Supplement: Supplementary file 3 — Additional file 3. Population – concept– context and inclusion and exclusion criteria. [file 12889_2025_22579_MOESM3_ESM.pdf]

Additional file 3.

Table. Population – concept – context and inclusion and exclusion criteria.

**Determinant**	**Term / definition**
(1) Participant	• Young people.
(2) Concepts	• ART.<br>• Medication adherence.
(3) Context	• sub-Saharan Africa.
Inclusion criteria	• Research articles on primary research published from 2010.<br>• Young people from 10 to 24 years and includes the younger “adolescents” (10-19 years) and older “youth” (15-24 years).<br>• YPLHIV *including co-morbid conditions and on ART.<br>• ART or combination antiretroviral therapy.<br>• Perceptions and experiences on ART adherence and ART non-adherence.<br>• Research conducted in sub-Saharan Africa.
Exclusion criteria	• Review articles.<br>• Literature not published in the English language.<br>• Research articles published before 2010.<br>• Research focusing on children below 10 years and adults above 24 years is excluded.<br>• Young people not on ART.

Determinant	Term / definition
	• TasP, ARVs for PrEP, PEP and ARV-based microbicides.<br>• Research on measurements of adherence, retention and lost-to-follow up.<br>• Research conducted in countries outside sub-Saharan Africa.

* Factors surrounding adherence appear to be similar, *ART* Antiretroviral therapy. *YPLHIV* Young People Living with HIV, *ART* Antiretroviral therapy, *TasP* Treatment as Prevention, *ARV* Antiretroviral, *PrEP* Pre-exposure prophylaxis, *PEP* Post-exposure prophylaxis.
